# Development of Two-Dimensional Nanomaterials Based Electrochemical Biosensors on Enhancing the Analysis of Food Toxicants

**DOI:** 10.3390/ijms22063277

**Published:** 2021-03-23

**Authors:** Iruthayapandi Selestin Raja, Mohan Vedhanayagam, Desingh Raj Preeth, Chuntae Kim, Jong Hun Lee, Dong Wook Han

**Affiliations:** 1 BIO-IT Foundry Technology Institute, Pusan National University, Busan 46241, Korea; rajaselestin@gmail.com (I.S.R.); chuntae1122@gmail.com (C.K.); 2 CSIR-Central Leather Research Institute, Adyar, Chennai 600 020, India; msvedhanayagam@gmail.com; 3 Chemical Biology and Nanobiotechnology Laboratory, AU-KBC Research Centre, Anna University, MIT Campus, Chromepet, Chennai 600 044, India; preeth1905@gmail.com; 4 Department of Food Science and Biotechnology, Gachon University, Seongnam 13120, Korea; 5 Department of Cogno-Mechatronics Engineering, College of Nanoscience & Nanotechnology, Pusan National University, Busan 46241, Korea

**Keywords:** food safety, food toxicants, electrochemical biosensor, 2D nanomaterials

## Abstract

In recent times, food safety has become a topic of debate as the foodborne diseases triggered by chemical and biological contaminants affect human health and the food industry’s profits. Though conventional analytical instrumentation-based food sensors are available, the consumers did not appreciate them because of the drawbacks of complexity, greater number of analysis steps, expensive enzymes, and lack of portability. Hence, designing easy-to-use tests for the rapid analysis of food contaminants has become essential in the food industry. Under this context, electrochemical biosensors have received attention among researchers as they bear the advantages of operational simplicity, portability, stability, easy miniaturization, and low cost. Two-dimensional (2D) nanomaterials have a larger surface area to volume compared to other dimensional nanomaterials. Hence, researchers nowadays are inclined to develop 2D nanomaterials-based electrochemical biosensors to significantly improve the sensor’s sensitivity, selectivity, and reproducibility while measuring the food toxicants. In the present review, we compile the contribution of 2D nanomaterials in electrochemical biosensors to test the food toxicants and discuss the future directions in the field. Further, we describe the types of food toxicity, methodologies quantifying food analytes, how the electrochemical food sensor works, and the general biomedical properties of 2D nanomaterials.

## 1. Introduction

The variety and quantity of food have increased dramatically as the food industry and modern agriculture are developed. Meanwhile, food safety holds a significant socioeconomic impact creating awareness among consumers [1,2]. Food safety is generally threatened by some molecular species, including pesticides, veterinary drug residues, heavy metals, pathogens, and toxins [3,4,5]. The presence of excessive chemical and biological toxins in food represents a serious threat to food safety and public health and reduces the food industry’s profits [6,7,8]. There was an increasing demand for strict testing for food toxicants, which has led to intensive research in food sensors. The World Health Organization (WHO) estimates that foodborne illnesses predominantly affect underdeveloped nations’ economies and placed food safety among its top 11 priorities [9,10].

Earlier liquid chromatography-based methods, including high-performance liquid chromatography (HPLC) and high-performance liquid chromatography coupled with tandem mass spectrometry (HPLC/MS/MS), were used for the accurate quantification of toxins [11,12]. Although these methods have more reliability and accuracy, they require expensive laboratory facilities, complex pre-treatment processing of the sample, and skilled operators [13,14]. Due to these drawbacks, HPLC-based methods’ application is limited in the on-site analysis of toxins [15]. Researchers have developed various sensing techniques over the past two decades, such as colorimetric assays, fluorescence biosensors, competitive enzyme-linked immunosorbent assays, microfluidic immunoassays, surface plasmon resonance biosensors, and electrochemical biosensors for the analysis of toxins in food, water, and clinical samples [16,17,18,19]. Among these biosensing systems, electrochemical biosensors/transducers of food toxins have become powerful tools offering several advantages such as operational simplicity, high sensitivity, easy miniaturization, relatively low cost, and suitable on-site analysis [20,21,22]. This technique expedites the screening process of food contamination and enables the remedial measurements to be taken promptly to manage the problems related to foodborne ailments.

Nanotechnology-derived products have offered a wide range of material candidates to increase the stability, selectivity, and sensitivity of electrochemical sensors [23,24]. The nanomaterials applied in the food industry have beneficial properties, such as drug encapsulation and delivery, antioxidant and antimicrobial property, and food additives increasing the food products’ flavor and shelf-life [1,10,25]. The functional nanomaterials produce a combined effect on catalytic activity, signal transduction, and high specificity on recognizing different molecules in electrochemistry-based devices. Hence, the construction of active nanomaterial-modified electrodes is extensively applied for food safety measurement [26,27,28]. As the two-dimensional (2D) nanomaterials exhibit a larger surface area to volume, they have been preferential candidates in designing various biosensors [29,30,31]. In the present review, we focus on developing 2D nanomaterials-based biosensors to detect food contaminants in real sample analysis. Apart from that, we highlight different types of food toxicity, various electrochemical methodologies to determine food toxicants, and the mechanism of electrochemical sensors.

### 1.1. Description of Food Toxicants

**Figure 1:**
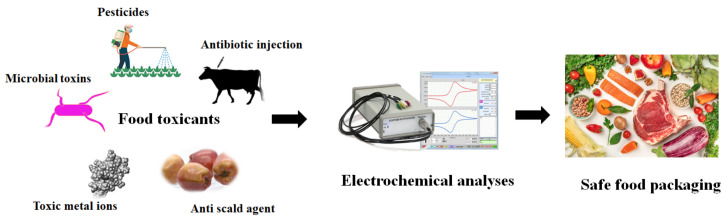
Ensuring food safety through electrochemical analyses of food toxic analytes from different sources has been shown schematically.

Food additives, chemical contaminants, and microbial contaminants are the three kinds of food analytes [32]. Food additives are included in food and drink items to produce desired color and flavor; however, overconsumption of additives can cause adverse effects to the human body [33,34]. The scientific reports reveal that a high caffeine dose can cause irritability, oversensitivity, and insomnia [35]. The primary public concern about food safety is to ensure strict control of the food additive concentration in food items geared toward growing children. Unlike the additives, the contaminants are not included in the food items intentionally [36]. Chemical contaminants, such as pesticides and veterinary drugs, significantly reduce food quality in food processing and storage. In general, the family of β-agonists, including ractopamine, cimaterol, clenbuterol, and salbutamol, is used to improve the carcass leanness in livestock species [37,38]. The scientific reports reveal that such β-agonists cause several potential hazardous effects such as cardiac palpitation, nervousness, tachycardia, muscle tremors, and confusion [39,40]. Bacterial pathogens such as *Salmonella* (31%), *Listeria* (28%), *Campylobacter* (5%), and *Escherichia coli* O157:H7 (3%) species are the causative agents of microbial contamination [41]. They trigger water and food-borne diseases threatening human health [42]. Electrochemical biosensors are sensitive to detect even trace amounts of food analytes due to their high specificity of biological reaction combined with electrochemical techniques. Electrochemical biosensors’ important characteristics are amenability to miniaturization, dynamic concentration range, instant response to the analytes, and stability at varying environmental factors such as temperature and pH [21,22]. Figure 1 demonstrates the measurement of different types of food toxic analytes using an electrochemical biosensor to ensure food safety.

### 1.2. Classification of Electrochemical Biosensors

**Figure 2:**
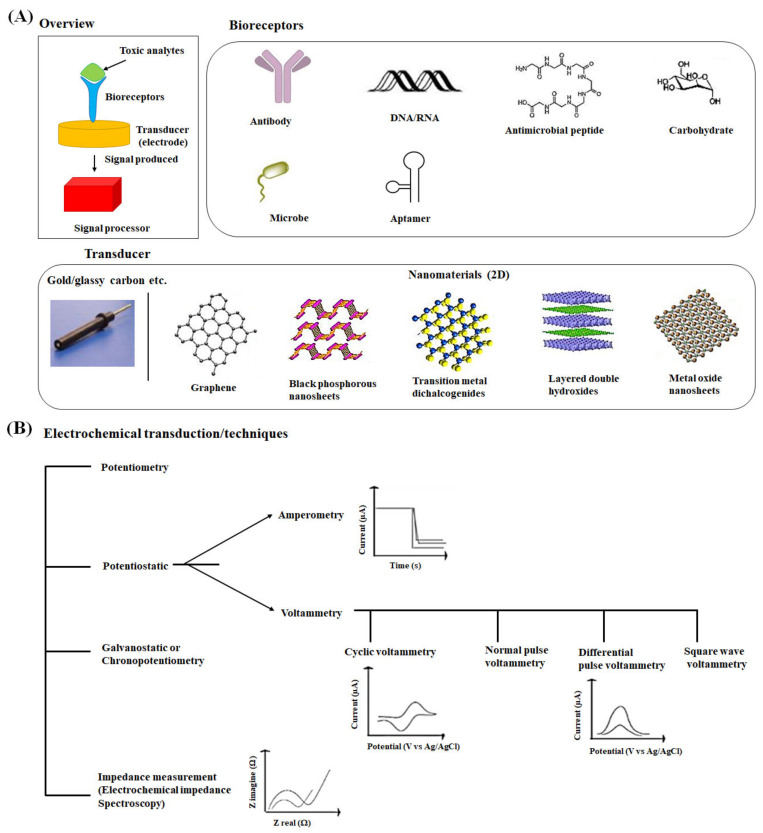
(**A**) Schematic illustration of a typical electrochemical food sensor and its various components, including bioreceptors, transducer, and signal processor [68]. The structure of two-dimensional nanomaterials used to fabricate the electrode surfaces in biosensors has been shown [69]. (**B**) Different types of electrochemical techniques have been presented [51].

We have exemplified the basic principle and the types of electrochemical biosensors in this section. The electrochemical biosensors work on the principle that an electrochemical signal is generated when the desired analyte is either oxidized or reduced upon the fixed or varying potential. The variation in electron fluxes is measured by the detector Figure 2A [43]. The biosensing surface may include any biological elements such as enzymes, antibodies, antigens, microorganisms, receptors, mammalian cells, and tissues immobilized on the transducer surface [44,45]. The biological elements bind the analyte (food toxicants) molecules selectively, and the surface of a transducer converts the event into a measurable electrical signal, voltage, or current [46]. Eventually, the electrochemical technique and the signal processor receive, magnify, and display the signal.

Depending on the biomolecular element’s detection mechanism, electrochemical biosensors can be categorized into biocatalytic and affinity sensors [47]. Biocatalytic sensors monitor the enzyme-target reaction to produce electroactive molecules. In contrast, affinity sensors, including aptasensors, immunosensors, and DNA sensors, observe the interaction between the bioreceptor and the target to generate a measurable signal [48]. A combination of signal transduction and biological receptor can also be described as an electrochemical affinity sensor. For instance, impedimetric immunosensors utilize impedance spectroscopy and antibodies [49]. The electrochemical sensors can be described as labeled or label-free sensors depending on the use of labels for improving their detection mechanism [50]. 

A variety of electrochemical techniques employed for the detection of toxic analytes can be classified into the following general categories, potentiostatic, galvanostatic, potentiometric, and impedimetric sensors (Figure 2B) [51]. In the potentiostatic method, the applied controlled potential to the electrochemical cell is converted into the current. In contrast, the current is applied to measure the potential in the galvanostatic method. When the cell potential is assessed under the near-zero current condition, it is known as potentiometric. The impedance method applies the cell’s potential, and the current response is measured to obtain impedance (complex resistance). The most common potentiostatic techniques in affinity electrochemical biosensors are amperometry and voltammetry, which apply a constant, scanning, or pulsing potential to a working electrode to measure the current [52].

Amperometry works on a fixed potential, whereas voltammetry examines a set of potential ranges to measure the current [53]. Voltammetric techniques, including cyclic voltammetry [54], normal and differential pulse voltammetry [37], and square wave voltammetry [55], have been extensively used for the analysis of various biological toxic analytes. Cyclic voltammetry is the commonly used electrochemical technique for the study of electroactive species. A characteristic cyclic voltammetric curve consists of a scanning trace from an initial potential to a switching potential followed by a reverse direction to the final potential [56]. Normal pulse voltammetry comprises a series of potential pulses with an increase in amplitude. The current response is evaluated near the end of each pulse when the interfering charging current decays away [51]. Differential pulse voltammetry scans the applied potential pulse of a constant amplitude through a fixed potential range and displays the difference in the two currents for the voltammogram [57]. In the square wave voltammetry technique, a symmetrical square wave is overlaid on a staircase waveform, and the difference in current between a forward and reverse pulse is computed. This technique has enhanced sensitivity for the analytes with faster scan rates than normal and differential pulse voltammetry methods [58]. 

Amperometric devices continuously measure the current resulting from redox reactions occurred by the electroactive species at a given potential. Clark oxygen electrodes, the simplest form of amperometric biosensors, produce current by reducing oxygen at a platinum working electrode in reference to a Ag/AgCl reference electrode [59]. Amperometric sensors can measure the analytes directly or indirectly. Direct amperometry provides an intimate relationship between the measured current and the products of the redox reaction. In contrast, indirect amperometry utilizes conventional detectors to measure the desired analytes’ metabolic substrate or product [59,60]. Amperometric techniques are mostly used to monitor various enzymatic reactions in a biocatalytic type biosensor.

When compared to the potentiostatic techniques, the usage of galvanostatic techniques in the biosensor is less. The chronopotentiometry method involves controlling the current between the working electrode and counter electrode and measuring the potential across them. It explores ion depletion at the membrane and sample interface and observes an inflection of the potential-time trace [61]. The potentiometric sensor investigates the potential difference between the working electrode and the reference electrode and displays the accumulation of charges at zero current created by the electrode surface [16]. The generation of potentiometric signals relies on a permselective transfer of analyte ions from the aqueous phase to the organic phase, which, in turn, creates a charge separation between the two phases [62]. Ion-selective electrodes and ion-selective field-effect transistors are the main components of potentiometric biosensors.

In the past two decades, electrochemical impedance spectroscopy (EIS) has been a robust technique as it can measure the analytes without using labels to produce a detectable signal [63]. One can measure the complex impedance, the sum of the real and imaginary components, by applying an alternating potential signal to the electrochemical cell varying a wide range of frequencies [64]. The impedance components, such as resistance and capacitance, provide information about the surface reactions and interface properties [65]. As the EIS measurement does not require sample preparation, it can be used for inline checking of food toxicants in the food supply chain. Scientific reports reveal that EIS can analyze food hazards in less than 1 h [66]. Further, the use of EIS in the food industry has been abundant, including for fruits, such as ripening of banana and dry matter of durian, for vegetables such as moisture content of carrots during drying, and changes in spinach tissue during heating. It helps evaluate discrimination of fresh and frozen-thawed chicken breast muscles, determination of the additives in natural juices, and bovine milk adulteration [67].

### 1.3. 2D Nanomaterials Improving Electrochemical Biosensors’ Performance

Nanomaterials used in electrochemical biosensors are mainly carbon-based nanomaterials [70,71,72], metal and metal oxide nanoparticles [73,74], and molecularly imprinted polymers [75,76]. The nature of the biosensing surface should exhibit prolonged stability for use and extended storage [77]. The nanomaterials incorporated in electrochemical biosensors improve response speed, sensitivity, and selectivity to meet the need for contaminant detection in food samples due to the nanomaterials’ unique physicochemical and electrical properties [78,79].

2D nanomaterials exhibit a large surface-to-volume ratio compared to other dimensional (0D, 1D and 3D) nanomaterials [80,81,82]. They provide numerous anchoring sites for analytes’ interaction, owing to their ultrathin planar nanostructure and large surface area, making them more suitable for sensor applications [83]. The 2D nanomaterials can display significantly higher conductivity than their 1D and 3D counterparts with their tunable electronic configuration and the resulting bandgap variation [84]. They express enhanced electrical properties leading to efficient signal transduction due to easily adjustable surface morphology [85]. The nanomaterials’ atomic-size thickness plays an important role in showing variations in fluorescence, magnetic permeability, and chemical reactivity [86,87]. 2D nanomaterials have better compatibility with metal electrodes with large lateral sizes. The literature reports reveal that 2D nanomaterials show excellent compatibility with ultrathin silicon channel technology, whereas 0D, 1D and 3D nanomaterials face difficulties with device integration, establishing electrical contacts, and device miniaturization, respectively [88,89]. Some of the 2D nanomaterials exhibit better mechanical strength and remarkable optical properties [90,91,92]. The desired physicochemical properties in 2D nanomaterials could be achieved by introducing defect engineering, doping, and fine-tuning of structural properties during preparation [93,94]. Overall, the rich surface chemistry, conductive property, fluorescence, and compatibility of 2D nanomaterials make them very suitable candidates for health and environmental monitoring [95,96]. 

2D nanomaterials include graphene family nanomaterials [97,98], MXene [99,100], transition metal dichalcogenides [101,102], single- elemental layered crystalline materials [103,104], and metal oxides [105,106]. Among them, graphene family nanomaterials have been explored widely by researchers for food sensing applications [107,108,109]. Graphene family nanomaterials, including graphene oxide (GO), and reduced GO (rGO), and single and multilayered graphene, have a hexagonal lattice structure involving a single layer of sp^2^-hybridized carbon atoms covalently bound together [110,111]. The number of layers in graphene, intercalated by weak van der Waals forces, influences their physicochemical properties in several applications [112,113]. The graphite containing more than 10 layers is subjected to different physicochemical methods such as mechanical cleavage and electrochemical exfoliation to produce graphene sheets with single or few layers [114]. Chemical vapor deposition is a broadly utilized bottom-up approach to synthesize precisely controlled nanographene [115,116]. Graphene has been widely used in several biomedical applications, including drug carriers [117], tissue regeneration [118], and cancer treatment [119,120] apart from biosensors [121,122]. Like graphene family nanomaterials, transition metal dichalcogenides, including molybdenum disulfide and molybdenum selenide, have shown remarkable physicochemical properties with biocompatibility, and have found significant advances in fabricating electrochemical biosensors [123,124,125]. Black phosphorus (BP) is composed of puckered lattice configuration and has exhibited more biocompatibility than other two-dimensional nanomaterials. Owing to unique semiconducting properties, anisotropic conductance, and larger hole mobility, they have been extensively applied in batteries and field-effect transistors. However, their application in electrochemistry is limited because of easy oxidization under normal conditions [126]. Cai et al. synthesized porous graphene-black phosphorous composite using a strong coherent coupling reaction. They used it to fabricate an electrochemical sensor to quantify bisphenol A, a food packaging material [127].

Among the metal oxides, manganese oxide (MnO_2_) has been widely used as electrode material for its numerous redox electrochemical reactions and low cost. Though the theoretical capacitance of MnO_2_ is high (1370 F g^−1^), its maximum electrochemical capacitance is around 250 F g^−1^ only. The attributed reasons are poor electrical conductivity and less charge storage practice [128,129]. To avail a better capacitance performance, Thangarasu et al. fabricated an electrochemical sensor based on a nanocomposite of MnO_2_/PANI/rGO to determine the level of methyl parathion, a pesticide [130].

Since 1987, the synthetic layered double hydroxides (LDH), known as anionic clays, have been used to modify electrodes. LDH materials have a lamellar structure with a high charge density of layers possessing intercalation properties. The net positive charge of the layer in LDH is maintained by the exchangeable anions intercalated between the octahedra forming sheets. The positively charged layers are the anchoring sites to immobilize the biomolecules, depending on their isoelectric point [131]. Shan et al. developed a LDH based electrochemical biosensor to immobilize polyphenol oxidase to determine toxic phenols [132].

MXenes, including 2D transition metal nitrides, carbides, or carbonitrides, are synthesized by etching out layer A selectively from parent MAX phases [133]. MAX phase contains alternating layers of M (transition metal) and A (group A element) with X (C or N) to form a closely-packed multilayer structure. Ti_3_C_2_ is the most studied material among the synthesized 20 different MXenes. Owing to layered morphology, large surface area, hydrophilicity, thermal stability, and high electrical conductivity, MXenes have found numerous applications in electrochemical biosensors [134]. Briefly, integrating 2D nanomaterials and their nanocomposites with electrochemical transducers in biosensor has a great potential to improve their analytical performance. And the scope of 2D nanomaterial-based electrochemical biosensors has constantly been expanding in the field of food safety. 

**Figure 3:**
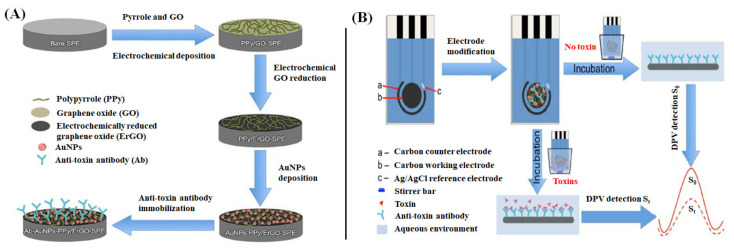
(**A**) Demonstration of step-by-step fabrication of the GO-based biosensor (antibody-Au NPs-polypyrrole/electrochemically reduced GO-screen printed carbon electrode) and (**B**) electrochemical immunosensing of the system employed for the detection of mycotoxins through DPV signals [135].

The architecture of the electrode surface can be controlled by fabricating with a high-density array of nanomaterials. While doing so, the nanomaterials’ intrinsic properties could be exploited at the electrode interface, enhancing the bioanalytical performance of a biosensor. For instance, Lu et al. have fabricated a GO-based electrochemical immunosensor to rapidly detect mycotoxins fumonisin B1 and deoxynivalenol [135]. The fabrication steps involve electrochemical deposition of polypyrrole (PPy)/GO nanocomposite film over a bare screen-printed carbon electrode. Subsequently, the GO was completely reduced to obtain PPy-electrochemically reduced graphene oxide (ErGO) nanocomposite film by cyclic voltammetry method. The resulting surface was drop-coated with AuNPs solution. Then, the modified electrode was immersed in 3-mercaptopropionic acid and EDC/NHS solutions to form an Au-S bond and activate the carboxyl groups, respectively. Finally, the electrode surface was immobilized with antibodies (Ab) by incubating with anti-toxins at pH 9.0 for 12 h, as shown in Figure 3A. The electrochemical immunosensing of the developed biosensor to target mycotoxin has been illustrated in Figure 3B. DPV peak currents are measured for a blank sample and the samples containing mycotoxins. The Ab-toxin interaction at the electrode surface results in a decrease in DPV current, which is proportional to the Ab vs. toxin concentration in the samples. Hence, the difference between the DPV peaks of blank and samples helps quantify the number of target toxins present in the sample.

## 2. Food Toxicant Analyses of 2D Nanomaterial-Based Electrochemical Biosensors

The role of 2D nanomaterials in electrochemical biosensors in sensing food toxicants has been summarized in Table 1. Various methodologies to detect food toxicity, linear range, and detection limit, and the value of recovery and repeatability in real sample analysis have also been described.

Eissa et al. developed a graphene-based voltammeter immunosensor to identify okadaic acid (OA) in spiked crustacean tissue extracts [136]. *Dinophysis* and *Prorocentrum*, as the most ubiquitous known dinoflagellates, produce OA, a lipophilic marine biotoxin, and accumulate it in shellfish [148]. When human beings consume OA-contaminated shellfish, OA inhibits protein phosphatase, such as PP1 and PP2A at the molecular level, and consequently causes a severe toxic effect known as diarrhetic shellfish poisoning [149,150,151]. Furthermore, it causes gastroabdominal disturbances, including vomiting, nausea, and diarrhea [152]. OA’s maximum limitation level in mussels is 160 µg kg^−1^ (EC no. 853/2004 15) [153,154,155]. To detect OA, they functionalized graphene-modified screen-printed carbon electrodes (GSPE) by electrochemical reduction of 4-carboxyphenyldiazonium salts in an acidic aqueous solution. Then, the OA was covalently bonded to the electrode surface using carbodiimide chemistry. The developed graphene-based immunosensor had a detection limit of OA 19 ng L^−1^ in PBS buffer, which is much lower than OA’s maximum limitation level in shellfish. The proposed electrochemical approach is a single-step and fast method to detect okadaic acid without using enzyme labeling and reduces both the assay’s cost and time. Further, this sensor works on a direct competitive assay to prove its specificity and sensitivity and has been validated using certified reference mussel samples showing good recovery%.

**Figure 4:**
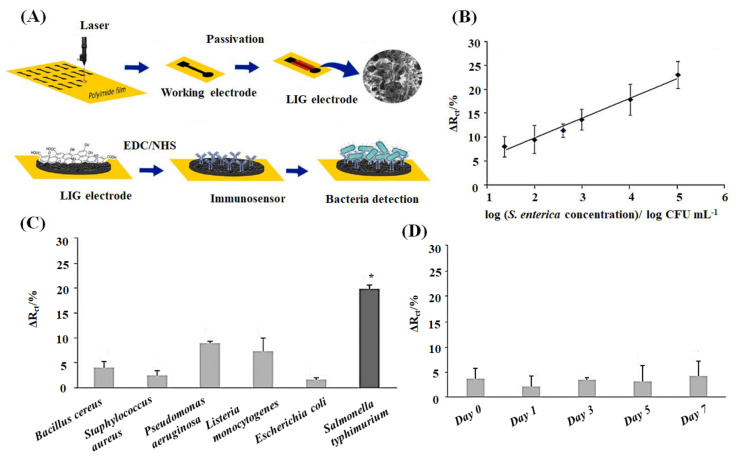
(**A**) Schematic demonstration of fabrication, biofunctionalization, and sensing of the LIG immunosensor. LIG is processed onto a polyimide sheet to create the working electrode, and subsequently, the electrode is passivated with lacquer. SEM image of the LIG surface is shown. The Salmonella antibodies are immobilized on the working electrode via carbodiimide cross-linking chemistry (EDC/NHS) to detect Salmonella microbes. (**B**) The linear calibration curve of charge transfer resistance change (ΔR_ct_) vs. *S. enterica* concentrations (generated from Nyquist plots of impedance spectra) in chicken broth. (**C**) ΔR_ct_ vs. different interferent bacterial species (10^4^ CFU mL^−1^) to show the specificity of the immunosensor. (**D**) Shelf-life test to investigate the stability of the immunosensors for seven days. All the data shown as mean ± SD, *n* = 3. * means significantly difference (*p* < 0.05) [137].

Soares et al. developed label-free laser-guided graphene (LIG) electrode functionalized with an antibody to electrochemically quantify the food-borne pathogen *Salmonella enterica* serovar *Typhimurium* [137]. According to data from the U.S. Food and Drug Administration (FDA) and the Centers for Disease Control and Prevention (CDC), *Salmonella enterica* is one of the leading causes of food-borne illness, which causes approximately 1.2 million illnesses and 450 deaths each year in the United States [156]. The LIG biosensors detected live Salmonella species in chicken broth at a linear range of 25 to 10^5^ CFU mL^−1^ with a low detection limit of 13 ± 7 CFU mL^−1^. Figure 4 showed the fabrication, functionalization, and sensing potential of the sensor against bacterial microbes schematically. The advantages of this sensor are low cost and disposable. It can be applied to in-field food processing facilities to trace the contaminants, crucial for successful commercialization. The shelf life of freeze-dried (−20 °C) immunosensors has been reported to be seven days. The estimated cost of the developed sensor is inexpensive, with $1.76 per device.

Some algae species, including *Ptychodiscus brevis*, produce BTX-2 (brevetoxin B) which results in neurotoxic shellfish poisoning (NSP) by consuming BTX-2 contaminated shellfish [157,158]. To detect brevetoxin B (BTX-2) in seafood, Tang et al. devised a practicable and straightforward magneto-controlled immunosensing platform. In this platform, guanine-assembled graphene nanoribbons (GGNR) were used as molecular tags on magnetic carbon paste electrodes. Monoclonal mouse anti-BTX-2 antibody was covalently bound to the electrode surface. The chemically modified bovine serum albumin-BTX-2 conjugated (BTX-2-BSA) with the GGNRs acts as the recognition elements. Under optimal conditions, the magneto-controlled immunosensor showed a dynamic concentration range at 1.0 pg mL^−1^ to 10 ng mL^−1^ of BTX-2. [138]. When this chemical immunoassay was carried out for 12 spiked samples with *Musculista senhousia, Sinonovacula constricta*, and *Tegillarca granosa* comparing with the commercialized ELISA method, there was no significant differences found between them, which proves the reliability and potential of the proposed immunosensor.

Bulbul et al. studied a non-enzymatic nanocatalyst approach to construct an electrochemical aptasensor that involves the contribution of nanoceria (nCe) tag and GO for the detection of Ochratoxin A (OTA) in corn samples [139]. The nCe labeled target analyte was captured by the immobilized aptamer on the GO modified electrode’s surface. The electrochemical signal was generated by the redox reaction between the species and the nCe tag. Subsequently, the GO layer amplified the signal increasing the sensitivity of the assay. The aptasensor showed a linear response to OTA in the range of 0.15–180 nM with a detection limit of 0.1 nM under optimal conditions. The reported biosensor found an enhancement in the target analyte’s sensitivity as the immobilized aptamer captures nCe labeled targets distinguishing them from non-label targets. The literature reports reveal that filamentous fungi of *Aspergillus* and *Penicillium* produce OTA. This low molecular weight mycotoxin is known to contaminate various food items, including dried fruits, cereals, cocoa, spices, beer, and wine [159]. The International Agency for Research on Cancer (Group 2B) informed that OTA contributes to cancer development being nephrotoxic, teratogenic, and immunosuppressive [160]. The European Union has stated that the regulatory limits for OTA in raw cereal grains, dried fruits, coffee products, and grape juice are 5 μg kg^−1^, 10 μg kg^−1^, 5 μg kg^−1^, and 2 μg kg^−1^, respectively (EC no. 123/2005) [139,161]. Wang et al. developed an electrochemical-assisted desorption method for the solid-phase extraction of metal ions (Pb^2+^) in tap water, mineral water, and beverage [140]. An array-like polyaniline nanofiber synthesized on the surface of graphene oxide (polyaniline-GO) acted as a well-ordered conducting sorbent. The adsorption/desorption process was accompanied by the changes of the as-prepared sorbent in cyclic voltammetry. The limit of detection was found as 0.04 μg L^−1^ under the optimal pH value. The proposed electrochemically assisted desorption method is simple, cost-effective, rapid, and eco-friendly and highlights that it does not require any elution to elute the target analyte. The sensor displays good anti-interference properties across various interference ions and has the potential to extract the target-analyte in the field of food safety control. Heavy metal ions, especially lead, are toxic and carcinogenic to the human body [162]. The World Health Organization has established a limit of 10 μg L^−1^ for Pb^2+^ ions in drinking water. According to the Environmental Protection Agency, the maximum criteria for Pb^2+^ ions in fresh water and saltwater were 65 μg L^−1^ and 210 μg L^−1^, respectively [140,163].

Mohammad-Razdari et al. developed a promising electrochemical method using an impedimetric aptasensor based AuNPs/rGO nanocomposite-modified pencil graphite electrode for the detection of tetracycline (TET) in milk samples with high reproducibility [141]. Gold nanoparticles (AuNPs) are widely used in electrochemical biosensors owing to their excellent electrical conductivity and catalytic property [164,165]. The combination of rGO and AuNPs makes the composite increase the electron transfer rate on the electrode surface and provide self-assembling sites for the aptamer DNA segment. While recording the transfer resistance, ΔR_ct_ (R_ct_ before and after aptamer) of various antibiotics, such as TET, streptomycin, penicillin G, and sulfadiazine, the results revealed that the sensor is more sensitive towards TET. This impedimetric biosensor is a promising method for the quantitative and qualitative measurement of TET in milk samples. The sensor is potentially employed in the early screening of milk samples, demonstrating a high reproducibility and stability (21 days). Further, the sensor can detect other antibiotics in various food items, including shrimp, meat, and fish. TET is commonly used to treat infectious diseases, like mastitis [166]. There are more possibilities to contaminate the food products like milk, eggs, meat, and chicken when the antibiotic is overused as an antibacterial and growth enhancer in veterinary medicine [167]. Consumption of such contaminated food items causes increased drug resistance in the human body [168]. The European Union has established the maximum residue level of TET in milk, meat, and eggs to be 220, 220 and 440 nM, respectively [169]. Hence, the quantitative measurement of TET in milk samples using a sensitive method becomes essential to protect human health. 

**Figure 5:**
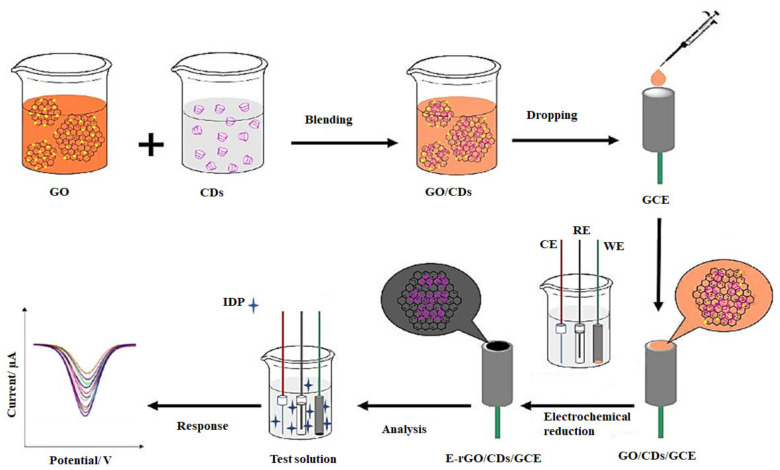
Schematic diagram demonstrating the preparation of food detection system containing electrochemically reduced graphene oxide/cyclodextrin modified glassy carbon electrode (E-rGO/CDs/GCE) to quantify the amount of imidacloprid (IDP) in test solution [142]. CE—counter electrode, RE—reference electrode, and WE—working electrode.

Imidacloprid (IDP), a typical neonicotinoid, is commonly used to control agricultural pests such as whiteflies, lepidoptera, and beetles and is one of the most used insecticides worldwide [170,171]. However, when a large quantity of IDT is absorbed by both vertebrates and invertebrates in the environment, the IDP residues cause a significant health risk to humans [172]. Zhao et al. fabricated an electrochemically reduced graphene oxide/cyclodextrin/glassy carbon electrode (E-rGO/CD/GCE) composite system for the detection of imidacloprid (IDP) residues in brown rice [142]. Initially, the complex of GO/CDs was prepared by simple stirring, and subsequently, GCE was modified using the complex by a drop-casting method. The oxygen-containing functional groups in GO were removed by an electrochemical reduction in PBS to obtain the desired electrochemically reduced composite system, as shown in Figure 5. To acquire the best signaling performance, they used three types of cyclodextrins (α-, β-, γ-CD) for analyzing sensor performance and found that α-CD had the best signal amplification for IDP. The developed sensor possessed long-term stability indicated by a more comprehensive linear range (0.5–40 μM) and a low detection limit (0.02 μM). The developed electrochemical sensor has advantageous properties, such as outstanding sensitivity, selectivity, stability, and reproducibility. The electrode fabrication via an electrochemical reduction approach is cost-effective and less time-consuming than the wet-chemical synthesis.

**Figure 6:**
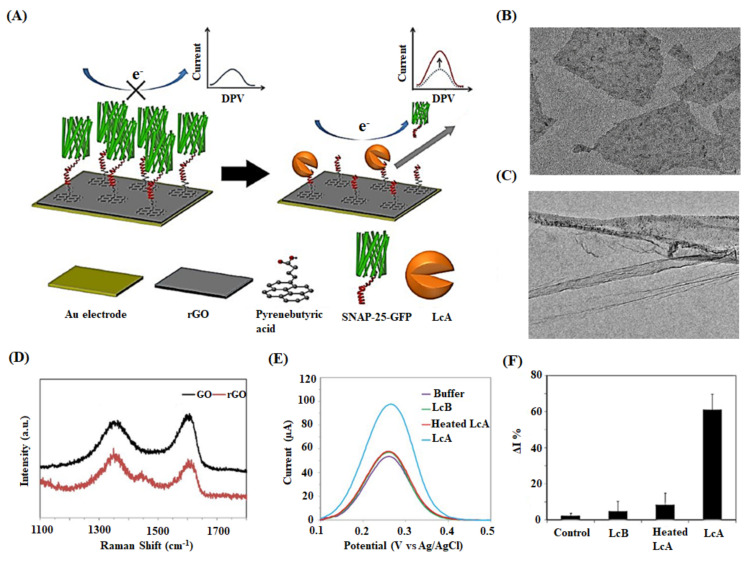
(**A**) Schematic diagram illustrating the detection mechanism of an rGO-based biosensor. SNAP-25-GFP peptide is immobilized on the rGO surface, which is previously conjugated with pyrenebutyric acid. The target BoNT-LcAs specifically cleave SNAP-25-GFP molecules, detaching them from rGO/Au electrode surface. The detection of enzymatic activity decreases the hindrance of redox probes transfer on electrodes resulting in increased electrochemical currents. (**B**) TEM image of rGO flakes and (**C**) rGO sheets with ripples and wrinkles. (**D**) Raman spectra of GO and rGO. (**E**) Specificity testing of control buffer and fresh BoNT-LcA, heated BoNT-LcA, and fresh BoNT-LcA at the concentration of 1 ng mL^−1^. (**F**) Relative DPV peak current change (ΔI%) for the same samples [143].

Chan et al. presented an rGO/Au electrode-based biosensor to detect botulinum neurotoxin serotype A light chain (BoNT-LcA) protease activity in milk samples [143]. The synaptosomal-associated protein 25-green fluorescent protein (SNAP-25-GFP) substrate was immobilized on the fabricated rGO/Au surface via a pyrenebutyric acid linker. BoNT-LcA cuts SNAP-25-GFP precisely at the cleavage sites to release the cut section from the electrode surface, detected by differential pulse voltammetry (DPV) (Figure 6). Though the proposed sensor witnessed the increased sensitivity, it had the drawback of non-specific adsorption of proteins in milk. The sensor was washed with Tween-20 after sample incubation to avoid other proteins’ interference in samples and the unwanted cleavage of SNAP-25-GFP. BoNT is a lethal neurotoxin secreted by *Clostridium botulinum* and can cause fatal paralytic illness botulism even in its low dose [173]. The researchers have identified seven serotypes (A–G) of botulinum toxins so far. Among them, BoNT/A has been reported to cause fatal food-borne botulism in human beings [174]. BoNT/A consists of a heavy chain and a light chain (LcA); however, the specific cleavage of SNAP-25 peptide is occurred by the LcA being responsible for the potential neurotoxicity [175]. Poo-arporn et al. developed a new disposable electrochemical sensor using a magnetic screen-printed electrode (MSPE) for the identification of ractopamine (RAC) in spiked real pork samples [144]. The electrode was modified with an iron oxide magnetic nanoparticle doping on reduced graphene oxide (Fe_3_O_4_/rGO) that promotes the electron transfer and raises the sensor’s sensitivity. The results of DPV showed a linear concentration range of 0.05–10 μM and 10–100 μM with a detection limit of 13 nM. The nanocomposite Fe_3_O_4_/rGO has promoted electron transfer, enhancing the sensitivity of the developed sensor. Further, the sensor is disposable and portable, with good reproducibility in on-site and real-time electroanalysis of the spiked pork samples. RAC is a β-adrenergic agonist, originally used to treat ailments including pulmonary disease and asthma [176]. Meanwhile, it has been illegally utilized as animal feed to reduce body fat deposition and improve protein accumulation [177]. Though the European Union has forbidden the employment of RAC in daily animal feeds, many countries, including China, are still allowing [178,179]. The drug residues accumulated in animal tissues can endanger consumer health, exhibiting symptoms such as cardiac palpitations, nervousness, muscular tremors, and tachypnea [180,181].

**Figure 7:**
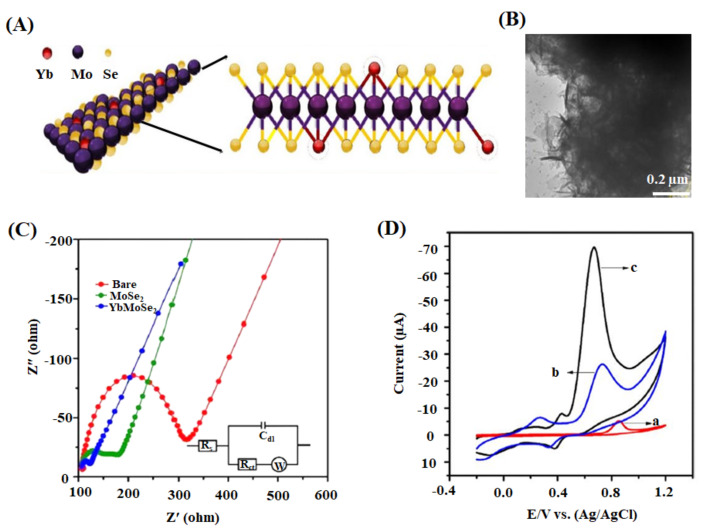
(**A**) Schematic of the proposed molecular packing structure and (**B**) HRTEM image of YbMoSe_2_. (**C**) Nyquist plot demonstrating the electrochemical performance of bare, MoSe_2_, and YbMoSe_2_ glassy carbon electrodes in 5 mM ferricyanide system in 0.1 M of KCl. The inset shows an equivalent circuit model (R_ct_—charge transfer resistance; C_dl_—double-layer capacitance; R_s_—solution resistance; W—Warburg impedance). (**D**) CV of bare GCE (a), MoSe_2_/GCE (b), and YbMoSe_2_/GCE (c) with 0.29 mM diphenylamine in N_2_ purged buffer at 50 mV s^−1^ [146].

Zhao et al. proposed a disposable electrochemical biosensor (Ti_3_C_2_T_x_ NSs/Au-Pd) to detect paraoxon organophosphorus pesticide in pear and cucumber samples [145]. The biosensor detected paraoxon with a linear concentration of 0.1–1000 μg L^−1^ and a low detection limit of 1.75 ng L^−1^. This enzymatic biosensor rapidly detects OPs exploiting superior conductivity and stability from the composite, MXene/Au-Pd. The screen-printed electrode (SPE) is disposable, and the nanoparticles (Au-Pd) are shape-controlled with desired catalytic activity. Organophosphorus pesticides (OPs) are compounds containing phosphorus elements, which control pests, plant diseases, and parasitic weeds [182,183]. When OPs are oxidized into highly toxic compounds, they cause more significant human health threats than the original compounds [145,184]. Ramaraj et al. developed a means to detect diphenylamine (DPA) in spike pear fruit by studying the electrocatalytic activity of YbMoSe_2_ modified glassy carbon electrodes (YbMoSe_2_/GCE) [146]. A high level of electrochemical activity of YbMoSe_2_/GCE was demonstrated with a low detection limit of 0.004 μM (Figure 7). DPA, a colorless aniline derivative, is used as a post-harvest anti-scald agent to prevent the decomposition of apples and pears during storage. Meanwhile, excessive consumption of DPA causes severe health issues to humans, such as bladder diseases, red blood cell damage, and hypertension [185,186]. Therefore, the European Union has proposed the daily acceptable level of DPA in fruits of about 10 mg kg^−1^ for pears and 5 mg kg^−1^ for apples [187,188]. The incorporation of Yb’s heterogeneous spin with MoSe_2_ generated the lattice distortion increasing the active sites, which helped for the high level of reproducibility, selectivity, and stability during the detection of target-analyte (DPA). The decreasing bandgap enabled an exceptional electronic conductivity and electrochemical activity in the proposed biosensor.

Xu et al. modified a glassy carbon electrode with black phosphorus nanosheets (BP NSs) and an aptamer to identify mycotoxin patulin (PAT) in spiked food samples [147]. The impedimetric assay measured PAT over a linear range from 1.0 nM to 1.0 μM with a detection limit of 0.3 nM. The electrode was further modified with gold nanoparticles to improve the sensor’s performance, which showed a more comprehensive linear range of 0.1 nM to 10.0 μM and a low detection limit of 0.03 nM. The larger surface area of BP NSs increased the loading of AuNP and aptamers on the electrode surface, effectively amplifying the biosensor’s signal. The AuNPs provided the anchoring sites of the aptamer to enhance the sensor’s efficiency of electron transport. However, the method had more time-consumption for the preparation of nanomaterials and the modification of electrodes. Some fungi, including *Aspergillus*, *Penicillium*, and *Byssochlamys*, produce patulin (4-hydroxy-4H-furo[3,2-c] pyran-2[6H]-one) as a secondary mycotoxin product [189,190]. The regulation by The Joint Food and Agriculture Organization/World Health Organization Expert Committee on Food Additives has determined the maximum daily level of PAT is 0.4 μg kg^−1^ by body weight [191].

## 3. Recent Advancements in Biosensors to Analyze the Food Toxicants

The potential revolution in consumer, healthcare, and manufacturing testing has made the global biosensor market worth over 10 billion dollars per annum and has been a burgeoning field of interdisciplinary research. However, an important barrier to biosensors’ widespread marketing is their cost, although many systems have been validated and proven at the concept level in the laboratory setting [192]. As there is downward pressure on costs, researchers are keen on developing different biosensors, such as multianalyte, flexible, hand-held, or computerized biosensors, without compromising their sensitivity and specificity [193]. This section is pertinent to all types of nanoparticles-based electrochemical biosensors but not limited to 2D nanomaterials-based sensors.

Regenerating biosensors is a recent technique to develop multianalyte biosensors enabling their reuse and reducing the cost per test. Regeneration has been achieved in amperometric and potentiometric sensors by overcoming the analyte and bioreceptor’s attractive forces. The contribution of enthalpy and entropy must be considered in thermodynamics, and the forces in the solvent environment can be altered using a regeneration buffer. The essential criteria for the successful regeneration of a biosensor are as follows: The signal loss between the interrogation cycles must be less than 5% showing the signal loss profile linear for accurate calibration, and more than 10 continual cycles must be achieved in restoring the baseline signal to < ±5% [194]. 

The development of hand-held devices connected with packing materials has enabled monitoring food quality throughout the entire supply chain and reported food spoilage possible to the consumers in commercial places such as supermarkets [195]. Hand-held devices, such as smartphones, are practical compact systems in conducting real-time testing. It is predicted that the usage of smartphones worldwide will increase by 58% from 2016 to 2022 [196]. The implementation of a piece of software into smartphones can be correlated with cellphones’ imaging capacity. When the biosensors analyze the food toxicants, the smartphones will gather the data and process them into readable information for the consumers. Flexible biosensors are being developed as wearable devices, such as smart wristbands, for emerging disposable biosensing applications. The wristband comprises a small battery, a flexible sensor array, and a flexible printed circuit board [197]. Consumers are using these kinds of biosensors for health monitoring. The same technique can be applied to in situ detection and long-term monitoring of toxic analytes in the food industry.

## 4. Conclusions

In the present review, we have discussed the recent development of 2D nanomaterial-based electrochemical biosensors in analyzing food toxicants. The studies have exposed 2D nanocomposites’ electrochemical analytical efficiency encompassing their sensitivity with a wide linear range and limit of detection and their real sample application with the salient findings of recovery and repeatability. Electrochemical biosensors provide sensitive qualitative and quantitative measurements of the analytes. The methods developed are inexpensive and time-saving compared with traditional analytical methods. These sensors involve in real-time and highly selective analyses without pre-concentration steps in many cases. Although these kinds of biosensors display remarkable advantages over traditional methods, there are still many difficulties developing a perfect biosensing technique to make them commercialized quickly: (1) Except for graphene family nanomaterials, only a few reports are available based on graphene-like 2D nanomaterials-based electrochemical food sensors. (2) The physicochemical properties of 2D nanomaterials play a pivotal role in determining their biosensing ability. But, in many kinds of literature, the researchers have failed to provide the parameters such as size and lateral thickness of the nanomaterials. (3) A comparative study in food toxic analysis among the 2D nanomaterials or with other dimensional nanomaterials should be carried out to bring out more effective compounds in the field. (4) The concentration of nanoparticles should be optimized to know the point at which the desired analytes attain saturation level during analysis. (5) Though electrochemical biosensors found advancement to detect food analytes with higher sensitivity, the analyte’s residence time with the electrochemical interface is still questionable. (6) The biological interaction of the analytes with various 2D nanomaterials should be discussed. The following comments can be suggested for future directions in this field: (1) Analyzing the electrochemical sensing potential of 2D nanomaterials following the thorough study of electrical conductivity, electrochemical conversion capability, and biomolecule immobilization capacity, dependent on size, shape, morphology, and defects of the nanoparticles. (2) Performing computational studies to have a deep insight into molecular interaction of food toxicants with 2D nanomaterials. (3) Determining 2D nanomaterial’s preferential selectivity with the similarly classified but differently molecular structured food toxicants. (4) Developing electrochemical sensors with microfluidics, microelectrode arrays, signal amplification, magnetic filtration, and antibody design to improve the target’s sensitivity and selectivity. (5) Achieving many more inexpensive and long storage devices without compromising the accuracy of the analysis. We hope that the researchers with interdisciplinary backgrounds will advance the field of electrochemical food sensors by signifying the importance of 2D nanomaterials considering the associated problems and the concerning suggestions.

## Figures and Tables

**Table 1:** Contribution of 2D nanomaterials in electrochemical biosensors in sensing food toxicants.

2D Nanomaterials and Composites	Transducer/Complex	Methodology	Food Contaminants	Linear Range; Limit of Detection	Real Sample Application	Recovery (%); Repeatability (%)	Remarks	**Study Authors**
Graphene	BSA/antibody/4-carboxyphenyl diazonium salt/GSPE	SWV	Okadaic acid/lipophilic marine biotoxin	~5000 ng L^−1^; 19 ng L^−1^	Shellfish extracts	89.2–104%; 5.8–10.9%	Single-step and rapid; reduced time and cost; enhanced sensitivity and specificity	Eissa et al. (2012) [136]
Multilayer graphene	LIG/multilayer graphene	EIS	*Salmonella enterica* serovar Typhimurium/food-borne pathogen	25 to 10^5^ CFU mL^−1^; 13 ± 7 CFU mL^−1^	Chicken broth	nd:nd	Low cost and disposable; shelf life for 7 days; inexpensive	Soares et al. (2020) [137]
Guanine-assembled graphene Nanoribbons (GGNRs)	Brevetoxin B -BSA-GGNRs	SWV	Brevetoxin B/neurotoxin	1.0 pg mL^−1^ to 10 ng mL^−1^; 1.0 pg mL^−1^	(Mollusk extracts)		Enhanced sensitivity equivalent to the commercialized ELISA method	Tang et al. (2012) [138]
					*Sinonovacula constricta*	94–112%; nd		
					*Musculista senhousia*	94–104%; nd		
					*Tegillarca granosa*	86–108%; nd		
GO	Bare/GO/EDC/aptamer/nanoceria labeled ochratoxin A	CV	Ochratoxin A/mycotoxin	0.15–180 nM; 0.1 nM.	Corn	92.5–96%; 3.1–4.3%	Enhanced sensitivity and selectivity	Bulbul et al. (2015) [139]
GO	Polyaniline-GO	FAAS and electrochemical assisted solid phase extraction	Lead (Pb^2+^)/toxic metal ions	ND;0.04 μg L^−1^	Tap water, mineral water, and beverage	nd: 0.14%	Simple and rapid; inexpensive and eco-friendly; exhibiting good anti-interference property	Wang et al. (2018) [140]
rGO	Aptamer-AuNPs-rGO-PGE	EIS	Tetracycline/antibiotic	1 × 10^−16^–1 × 10^−6^ M; 3 × 10^−17^ M	Cow milk	94.2–96.1%; 6.3–6.5%	Early screening; high reproducibility; stability for 21 days	Mohammad-Razdari et al. (2020) [141]
					Sheep milk	92.8–98.4%; 4.3–7.6%		
					Goat milk	95.7–97.1%; 4.4–8.4%		
					Water buffalo milk	97.7–102.1%; 9.2–10.2%		
rGO	rGO/α-cyclodextrin/GCE	LSV	Imidacloprid/neonicotinoid	0.5–40 μM; 0.02 μM	Brown rice	92.0–98.7%; 1.4–3.8%	Excellent sensitivity, selectivity, stability, and reproducibility; cost-effective and less time-consumption	Zhao et al. (2020) [142]
rGO	rGO/Au/pyrenebutyric acid/SNAP-25-GFP	DPV	Botulinum neurotoxin serotype A/neurotoxin	1 pg/mL to 1 ng/mL; 8.6 pg/mL	Skimmed milk	nd: nd	Increased sensitivity; non-specificity	Chan et al. (2015) [143]
Fe_3_O_4_/rGO	Fe_3_O_4_/rGO/MSPE	DPV	Ractopamine/β-adrenergic agonist	0.05–10 and 10–100 μM; 13 nM	Spiked real pork	90.13–109.63%; 1.81–5.03%	Enhanced sensitivity; portable; good reproducibility	Poo-arporn et al. (2019) [144]
Ti_3_C_2_T_x_ NSs/Au- Pd NPs	SPE/Ti_3_C_2_T_x_ NSs/Au-Pd/GA/AChE	Amperometry	Paraoxon/organophosphorus pesticides	0.36‒3634 nM; 6.36 pM	Pear	91.15–111.02%; 2.91–6.37%	Desired catalytic activity; rapid; superior conductivity and stability	Zhao et al. (2018) [145]
					Cucumber	87.93–110.82%; 1.08–5.89%		
YbMoSe_2_	YbMoSe_2_/GCE	DPV	Diphenylamine/anti-scald agent in fruits	0.01–80 μM; 0.004 μM	Spiked pear fruits	99–110%; 2.09–2.34%	Increased active sites and decreased bandgap; high reproducibility, stability, and selectivity	Ramaraj et al. (2019) [146]
BP NSs	Aptamer-BP NSs/GCE	EIS	Patulin/mycotoxin	1× 10^−3^–1 µM; 0.03 × 10^−3^ µM	Apple juice	97.3–104.6%; 2.8–4.2%	Effective amplification of biosensor’s signal; enhanced sensitivity; more time-consumption	Xu et al. (2019) [147]
Au NPs-BP NSs	Aptamer-Au NPs-BP NSs/GCE	EIS	Patulin/mycotoxin	0.1 × 10^−3^–10 µM; 0.03 × 10^−3^ µM	Apple juice	96.2–104.0%; 2.4–3.8%	Effective amplification of biosensor’s signal; enhanced sensitivity; more time-consumption	Xu et al. (2019) [147]

nd = no data available.

## Data Availability

No new data were created or analyzed in this study. Data sharing is not applicable to this article.

## Abbreviations

GCE	Glassy carbon electrode
SPE	Screen-printed electrodes
GSPE	Graphene modified screen-printed carbon electrode
PGE	Pencil graphite electrode
MSPE	Magnetic screen-printed electrode
LIG	Laser-induced graphene
EIS	Electrochemical impedance spectroscopy
LSV	Linear sweep voltammetry
DPV	Differential pulse voltammetry
CV	Cyclic voltammetry
SWV	Square wave voltammetry
FAAS	Flame atomic absorption spectrometry
BP NSs	Black phosphorus nanosheets
GO	Graphene oxide
rGO	Reduced graphene oxide
Ti_3_C_2_T_x_	Titanium carbide MXene nanosheets
YbMoSe_2_	Ytterbium-doped molybdenum selenide
Fe_3_O_4_	Iron oxide
AChE	Acetylcholinesterase
Au-Pd	Gold-palladium
CFU	Colony forming unit
EDC	1-(3-Dimethylaminopropyl)-3-ethylcarbodiimide hydrochloride
SNAP-25	Synaptosomal-associated protein-25
GFP	Green fluorescent protein
BSA	Bovine serum albumin
